# Rethinking Evidence Generation for Vaccine Safety in Pregnancy: Beyond Randomized Trials Across the Immunization Lifecycle

**DOI:** 10.3390/vaccines14100847

**Published:** 2026-09-25

**Authors:** Médéa Locquet, Jean-Michel Dogné

**Affiliations:** 1 Department of Biomedical Sciences, Clinical Pharmacology and Toxicology Research Unit, Namur Research Institute for Life Sciences (NARILIS), Faculty of Medicine, University of Namur, 5000 Namur, Belgium; 2 Department of Pharmacy, Clinical Pharmacology and Toxicology Research Unit, Namur Research Institute for Life Sciences (NARILIS), Faculty of Medicine, University of Namur, 5000 Namur, Belgium

**Keywords:** maternal immunization, vaccine safety, pregnancy, pharmacovigilance, real-world evidence, post-authorization safety study, pharmacoepidemiology, causal inference

## Abstract

Maternal immunization protects pregnant individuals and infants. However, evidence on vaccine safety during pregnancy remains variable across vaccine products and maternal, fetal, neonatal, and infant outcomes. Debate has often focused on whether pregnant individuals should be included in randomized controlled trials, overlooking the complementary roles of other study designs. Trials are indispensable for assessing immunogenicity, reactogenicity, efficacy, and common short-term outcomes, but are rarely powered to exclude modest increases in uncommon obstetric, congenital, neonatal, or delayed childhood outcomes. This Perspective examines a coordinated approach to maternal vaccine safety assessment before and after authorization, including randomized trials, spontaneous reporting, pregnancy vaccine exposure registries, linked maternal-infant databases, near-real-time surveillance, and international research networks. Lessons from influenza, pertussis, COVID-19, and maternal respiratory syncytial virus vaccination show why authorization should initiate sustained pregnancy-specific safety monitoring. We discuss the limitations of real-world evidence, including confounding, healthy-vaccinee effects, gestational timing, conditioning on live birth, outcome misclassification, and background rate estimation, as well as methods to strengthen causal interpretation. Regulators and vaccine developers should prespecify pregnancy-specific post-authorization studies, harmonize outcome definitions and exposure windows, and distinguish the absence of a detected signal from the exclusion of a clinically relevant risk. Coordinated surveillance is essential to provide timely, precise, and clinically meaningful evidence for pregnant individuals and their children.

## 1. Introduction

Maternal immunization can confer two complementary forms of protection: direct protection of the pregnant individual against vaccine-preventable disease and indirect protection of the fetus or young infant through transplacental transfer of maternal antibodies. These effects coexist with pregnancy-specific safety questions that span maternal, fetal, neonatal, and childhood time horizons. These benefits underpin established tetanus, influenza, and pertussis programs and, more recently, coronavirus disease 2019 (COVID-19) and respiratory syncytial virus (RSV) vaccination in selected settings [1,2,3]. Vaccination nevertheless occurs during a biologically dynamic period in which miscarriage, hypertensive disorders, preterm birth, fetal growth restriction, congenital anomalies, stillbirth, and neonatal morbidity arise independently. Temporal associations can therefore raise concern, while weak study designs can create false signals or false reassurance.

Pregnancy vaccine safety gaps are often attributed to the historical exclusion of pregnant individuals from pre-authorization randomized controlled trials (RCTs), which raises legitimate ethical and equity concerns. Yet inclusion alone cannot resolve them: many decision-relevant outcomes are uncommon (e.g., stillbirth, specific major congenital anomaly phenotypes, autism spectrum disorder), heterogeneous, or diagnosed only after prolonged infant follow-up. Even an RCT involving several thousand pregnancies may estimate common outcomes and detect large imbalances, but would remain unable to exclude modest increases in specific congenital anomalies, stillbirth, or developmental disorders. The central question is which combination of designs can address a defined safety concern with adequate power, valid comparators, appropriate follow-up, causal validity, and a proportionate burden for pregnant participants and, where required, their infants during follow-up [4]. This statistical limitation is not unique to pregnancy: pre-licensure vaccine RCTs in any population are generally unable to exclude very rare adverse events, while pregnancy additionally requires gestationally specific exposure assessment and maternal-fetal-infant follow-up [5,6,7].

This Perspective proposes a pregnancy-specific framework for vaccine safety assessment across the immunization lifecycle, integrating recent regulatory developments, established maternal immunization programs, lessons from COVID-19 vaccination, and emerging post-authorization evidence on maternal RSV vaccination. Rather than considering randomized trials and observational studies as competing sources of evidence, we present a lifecycle evidence architecture in which RCTs, pharmacovigilance, pregnancy registries, linked healthcare databases, active surveillance, and advanced causal inference methods provide complementary evidence with different strengths and limitations for different pregnancy safety questions. Throughout this Perspective, the term lifecycle evidence architecture refers to the coordinated generation and integration of complementary evidence across vaccine development and post-authorization surveillance. Within this framework, post-authorization evidence generation is not simply a continuation of pre-authorization evaluation, but an essential step in reducing uncertainty about rare, delayed, and population-level outcomes that cannot be adequately addressed before vaccine approval [7,8,9,10,11].

### Evidence Identification and Narrative Synthesis

This Perspective was informed by a targeted narrative search of PubMed/MEDLINE, supplemented by current regulatory sources (ICH, EMA, FDA/CDC, and WHO) and by reference list screening of major publications. The evidence search was updated through 19 September 2026. Search terms included maternal or pregnancy vaccination, vaccine safety, pharmacovigilance, pregnancy exposure registries, linked maternal-infant databases, active or sequential surveillance, congenital anomalies, pregnancy loss, preterm birth, target trial emulation, and post-authorization safety studies. The search was not date-restricted; we prioritized current guidance and recent methodological and vaccine-specific evidence while retaining seminal earlier studies when directly relevant.

Priority was given to references including pregnancy-specific RCTs, large population-based comparative studies, validated methodological studies, systematic reviews or meta-analyses, and current regulatory guidance. Evidence was synthesized narratively by safety question, outcome frequency, gestational timing, required follow-up, and principal sources of bias and limitations. The objective was to develop a methodological narrative review rather than an exhaustive systematic review. Accordingly, we did not undertake a formal study-level risk-of-bias assessment or quantitative meta-analysis.

## 2. Why Vaccine Safety in Pregnancy Is a Distinct Evidence Concern

Vaccine safety in pregnancy cannot be treated as a conventional single-patient adverse-event problem. Benefits and risks may differ for the pregnant individual, fetus, neonate, and child. Maternal reactogenicity may be acceptable when severe infection is prevented; fever may require gestation-specific assessment; and infant benefit depends partly on the vaccination-to-delivery interval. Exposure windows, mechanisms, outcomes, and follow-up must therefore be specified separately rather than collapsed into a generic category of “pregnancy safety” [2,4,6].

Outcome frequency determines the evidence required. Common local and short-term systemic reactions can be estimated in hundreds of RCT participants. Phase 3 RCTs may also estimate preterm birth and hypertensive disorders, but often remain imprecise for modest clinically relevant differences. Major congenital anomalies are uncommon, and individual phenotypes may occur in fewer than 1 per 1000 or 1 per 10,000 births [12]. Their evaluation therefore requires very large population-based datasets and appropriate follow-up.

Restriction to live births can further bias inference because vaccination, infection, miscarriage, termination after prenatal diagnosis, stillbirth, and loss to follow-up jointly determine inclusion. A null RCT finding for a rare outcome, therefore, carries different evidentiary weight from a null finding for reactogenicity [13,14].

Gestational-age-specific background rates are essential for interpreting events that occur after vaccination, because pregnancy outcomes will occur coincidentally even without a vaccine effect. Pregnancy outcomes vary by maternal age, parity, comorbidity, socioeconomic context, season, healthcare access, infection burden, and ascertainment. With millions of doses administered, adverse events will occur by chance after vaccination. Observed-to-expected analyses can rapidly contextualize such events [15], but require population-, calendar-, and gestational-age-matched rates. Historical or geographically mismatched rates, or rates pooled across gestation, could be inappropriate for vaccines offered within narrow windows.

Communication of pregnancy vaccine safety findings should reflect the level and the maturity of the evidence. Pregnant individuals and clinicians often make decisions under uncertainty, and preliminary findings can strongly affect confidence. “No signal detected” in an underpowered RCT does not rule out risk, whereas an unadjusted numerical imbalance does not establish causality. Communication should report the design, detectable effect, confidence intervals, residual biases, and replication evidence. For example, early COVID-19 pregnancy data were necessarily descriptive before larger comparative studies became available, whereas the RSVpreF preterm-birth imbalance illustrates why a numerical RCT imbalance should be communicated as a signal requiring evaluation rather than as established harm [16,17,18,19,20,21,22,23,24,25,26].

## 3. What RCTs Can and Cannot Establish for Vaccine Safety in Pregnancy

RCTs are indispensable to maternal vaccine development because randomization balances baseline factors and enables standardized assessment of efficacy, immunogenicity, reactogenicity, maternal adverse events, obstetric outcomes, and infant outcomes. They can also inform the optimal gestational window by integrating immune response, transplacental antibody transfer, pathogen circulation, and feasibility [6,21,27,28].

These strengths do not overcome constraints imposed by outcome frequency, multiplicity, and follow-up. Maternal vaccine RCTs are usually powered for maternal or infant efficacy, not to exclude small increases across multiple rare outcomes. They can identify an imbalance requiring investigation but cannot reliably distinguish a small causal effect from chance. Follow-up also often ends within the first year of life, limiting assessment of delayed developmental or immune-mediated outcomes [5,7].

The ethical case for greater inclusion of pregnant individuals in RCTs should be matched by scientific rigor [4]. Inclusion is particularly informative for immunogenicity, efficacy, pharmacology of novel platforms, reactogenicity, common short-term outcomes, and incident pregnancies. However, a small subgroup of pregnancies without clearly defined questions, adequate power, standardized outcomes, and continued infant follow-up is unlikely to generate decision-relevant evidence. RCT protocols should specify which questions RCTs can answer and which uncertainties require post-authorization safety studies (PASS) [4].

The value of an RCT should therefore be judged by its fit to the safety question, precision, follow-up, and endpoint validity rather than by a fixed evidence hierarchy. RCTs are most informative for efficacy, immunogenicity, reactogenicity, and common short-term outcomes; linked cohorts and registries for comparative obstetric and neonatal risks; population-based pregnancy and congenital-anomaly data for rare phenotypes; and longitudinal maternal-child linkage for delayed outcomes. Appraise evidence from each source for comparator validity, exposure timing, outcome validation, major sources of bias, precision, and independent replication [6,7].

A proportionate approach should replace categorical exclusion. Early development should use platform knowledge, developmental and reproductive toxicology, and other non-clinical evidence to identify plausible maternal, fetal, and neonatal risks [29]. Include pregnant individuals in late-stage RCTs when anticipated use, disease burden, and the totality of evidence support a favorable benefit-risk balance. Participants who become pregnant should remain under protocol-defined follow-up, with further dosing stopped only when clinically indicated, and consent should explain both the value and limits of participation [29]. RCT planning should be prospectively aligned with pregnancy registries and pharmacoepidemiologic studies, using consistent exposure windows and standardized maternal, fetal, and infant outcomes to address residual uncertainty after authorization (Table 1) [4].

## 4. Regulatory Evolution Toward Vaccine Lifecycle Evidence During Pregnancy

Regulatory science increasingly treats pregnancy as a prespecified evidence domain when use during pregnancy or exposure among individuals of childbearing potential is anticipated. The draft International Council for Harmonization (ICH) E21 guideline recommends a structured, weight-of-evidence approach to determine when and how to include pregnant and breastfeeding individuals in clinical trials [8]. Relevant considerations include intended use, disease severity, developmental and reproductive toxicology, accumulated human experience, anticipated exposure, and scientific objectives. E21 does not require RCTs for every pregnancy outcome; it replaces default exclusion with prospective, proportionate evidence planning without prescribing the specific architecture proposed in this Perspective [8].

Complementing the ICH E21 framework, the final European Medicines Agency (EMA) Good Pharmacovigilance Practices (GVP) Product- or Population-Specific Considerations III extends this approach after authorization [9]. It recognizes that pregnancy data are often limited at approval and may require additional collection even without a pre-authorization concern. The guidance addresses gestational timing, follow-up of exposed pregnancies, individual case reports, risk management plans, periodic safety update reports, post-authorization safety studies (PASS), and long-term child outcomes. For vaccines, these provisions operate alongside GVP guidance for prophylactic vaccines, including exposure estimation, observed-to-expected analyses, batch and programmatic factors, and rapid signal evaluation [9]. The advantage of planned post-authorization collection is the ability to address rare outcomes, broader populations, and longer latency with substantially greater precision. The trade-off is greater susceptibility to confounding, exposure and outcome misclassification, and incomplete ascertainment than in randomized studies. Post-authorization evidence should therefore complement, not retrospectively substitute for, avoidable pre-authorization evidence gaps [7,9].

Together, these guidelines support a lifecycle evidence architecture in which pregnancy-specific evidence is generated prospectively and refined continuously throughout vaccine development and post-authorization use. Before authorization, developers should define the pregnancy-specific questions, smallest clinically meaningful risks to detect or exclude, data sources, comparators, validation procedures, and communication plans, and incorporate them into late-stage development and the risk management plan [8,9]. In practice, pregnancy should be treated as a planned evidence domain when maternal use is intended or pregnancy exposure is reasonably anticipated, with the intensity and timing of evidence generation proportional to expected exposure and clinical need. ICH E21 and EMA GVP provide the regulatory principles described above; the lifecycle evidence architecture proposed here is an authors’ methodological synthesis, not a regulatory standard. Its additional elements are the explicit question-to-design mapping, bias and sensitivity-analysis planning, and rules for integrating discordant evidence. Figure 1 and Figure 2 and Table 2 and Table 3 therefore present our proposed synthesis informed by, rather than reproduced from, existing guidance [7,8,9].

Evidence requirements should reflect the exposure context. Vaccines indicated for pregnancy require planned evaluation of maternal, fetal, neonatal, and infant outcomes; vaccines used after an individualized benefit-risk assessment require evidence tailored to the clinical setting [8,9]. Inadvertent exposure to vaccines that are not routinely recommended or are generally contraindicated in pregnancy, including live attenuated vaccines [31], requires focused follow-up of gestational timing, maternal immune status, fetal or congenital infection, pregnancy outcome, and congenital anomalies [8,9]. A recommendation against use based on theoretical concern should not be interpreted as evidence of fetal harm. For vaccines intended for populations in which pregnancy exposure is not reasonably anticipated (e.g., an older-adult-only vaccination indication), pregnancy-specific pre-authorization RCTs would not be proportionate. An incidental-exposure follow-up plan should instead be calibrated to plausible use [8]. For vaccines generally contraindicated in pregnancy, routine deliberate pregnancy exposure in trials is not justified solely to close a theoretical safety gap. Systematic follow-up of inadvertent exposures, pregnancy exposure registries, and population-based linkage can provide product-specific evidence. A systematic review of live attenuated vaccine exposures found no overall association with miscarriage, stillbirth, malformations, prematurity, or neonatal death, although certainty was very low and smallpox vaccine represented an important exception [31].

Despite these regulatory advances, methodological guidance for comparative post-authorization vaccine studies in pregnancy remains less developed than for pregnancy pharmacoepidemiology more broadly. Although initiatives such as the Global Alignment of Immunization Safety Assessment (GAIA) [37] have standardized case definitions and safety data collection, substantial heterogeneity persists in exposure definitions, comparator selection, outcome ascertainment, follow-up, and reporting. A shared protocol should be understood as a modular analytic specification rather than a single centrally enforced protocol. Within federated networks, common definitions, data models, and analytic code can be implemented locally under each data partner’s governance, with aggregate outputs synthesized across sites; permissions, funding, and data sharing agreements remain network- and study-specific [10,11,29]. Recent pregnancy-pharmacovigilance reporting recommendations further support standardized exposure and outcome definitions and transparent reporting of uncertainty [47].

## 5. Lessons from Maternal Immunization Programs

### 5.1. Influenza and Pertussis Vaccination

Maternal influenza and tetanus, diphtheria, and acellular pertussis (Tdap) programs show how safety evidence accumulates across complementary designs. Influenza RCTs established immunogenicity and efficacy in pregnant individuals and infants and standardized assessment of common short-term maternal, obstetric, and neonatal outcomes [27,28]. Their size, however, was insufficient to rule out modest increases in rare obstetric, congenital, or infant outcomes; observational studies, surveillance, and meta-analyses subsequently broadened the evidence across seasons, populations, and settings. In a Bangladesh trial, maternal influenza immunization reduced laboratory-confirmed influenza in infants by 63% and febrile respiratory illness in mothers by 36%; in a South African trial, efficacy against laboratory-confirmed influenza was approximately 50% in HIV-uninfected pregnant participants and their infants [27,28]. These trials demonstrated the value of randomization for efficacy and common outcomes while also illustrating why much larger post-authorization populations are required for rare safety outcomes.

For Tdap, linked databases and national observational studies evaluated obstetric and birth outcomes, major congenital anomalies, repeated tetanus-containing vaccination, and longer-term infant outcomes in populations far larger than feasible RCTs [48,49]. Consistency across countries, data sources, and analytic approaches provided stronger reassurance than any single pre-authorization study, illustrating that confidence develops through replication, standardized outcomes, and cumulative precision. These studies did not identify increased risks across the major obstetric and birth outcomes assessed, providing complementary evidence from routine care populations [48,49].

Important gaps remain for concomitant or closely spaced vaccination, underrepresented populations, and outcomes beyond infancy. Maternal antibodies can attenuate infant responses to subsequent vaccination, a phenomenon known as blunting [50,51]. Lower post-primary antibody concentrations have been reported for selected pertussis and coadministered antigens, with effects varying across maternal and pediatric formulations [52]. Although primarily an immunogenicity concern, its clinical significance remains uncertain, and established maternal programs have shown no increase in pertussis disease. Infant follow-up should therefore distinguish biomarker differences from clinical harm and account for the pediatric vaccination schedule.

### 5.2. COVID-19 Vaccines: Rapid Generation of Real-World Safety Evidence

The COVID-19 pandemic exposed the consequences of large-scale vaccination with limited pregnancy-specific RCT data. Pregnant individuals faced increased risks of severe disease and adverse obstetric outcomes but were largely excluded from pivotal SARS-CoV-2 vaccine RCTs. Initial recommendations therefore relied on platform knowledge, developmental and reproductive toxicology, incident pregnancies, and an evolving benefit-risk assessment. This uncertainty complicated early clinical and policy communication.

After rollout, pregnancy-specific safety evidence accumulated rapidly through overlapping systems. The United States V-safe pregnancy registry and Vaccine Adverse Event Reporting System (VAERS) provided early descriptive reassurance, but voluntary participation, selective reporting, incomplete follow-up, and weak comparators limited inference [16]. The Vaccine Safety Datalink and other linked databases then evaluated spontaneous abortion, preterm birth, fetal growth, stillbirth, and neonatal outcomes using defined gestational windows and concurrent comparators [17,18,19]. Larger population-based studies, including first-trimester exposure and a sibling-matched analysis, assessed congenital anomalies with greater precision and additional control for familial confounding [20].

The sequence matters. Early descriptive findings were preliminary; comparative studies better addressed confounding and selection, and replication across countries reduced reliance on a single system. COVID-19, therefore, supports a planned evidence sequence: immediate passive and active surveillance, rapid observed-to-expected and sequential analyses, prespecified comparative cohorts, and long-term maternal-child linkage for delayed outcomes. Communication should evolve with the design, precision, and maturity of the evidence.

### 5.3. Maternal RSV Vaccination: Signal Detection and Refinement

Maternal RSV vaccination shows why RCTs and post-authorization evidence must be interpreted together. A phase 3 RCT of the bivalent prefusion F vaccine demonstrated efficacy against severe RSV-associated lower respiratory tract illness in infants [21], but a non-significant numerical imbalance in preterm birth prompted regulatory scrutiny and contributed to restriction of the authorized gestational window in the United States. Such an imbalance can appropriately trigger precaution and prespecified post-authorization evaluation without being interpreted as proof of causality. In a separate phase 3 program, development of the RSVPreF3-Mat candidate was stopped after a higher incidence of preterm birth in the vaccine group [22], underscoring that safety findings are product-specific and should not be generalized solely from the antigen target or platform.

Post-authorization studies of the authorized RSVpreF product, including health-system cohorts, national analyses, electronic health record studies, and sequential surveillance, have not demonstrated a consistent increased risk of preterm birth to date within the authorized gestational window [23,24,25,26,43]. A recent Vaccine Safety Datalink target-trial emulation likewise found no higher risk of preterm birth, stillbirth, or small-for-gestational-age birth, while an association with hypertensive disorders of pregnancy warrants continued evaluation [43]. These findings are specific to RSVpreF and should not be extrapolated to RSVPreF3-Mat. As with other early post-authorization estimates, selective uptake, healthy-vaccinee effects, narrow eligibility, antenatal-care differences, residual confounding, and coding variation remain relevant limitations.

The RSV case illustrates a stepwise regulatory approach: the RCT imbalance informed labeling, gestational-window restrictions, and post-authorization studies; early surveillance assessed whether a large excess was emerging; and comparative studies improved precision and control for confounding. Findings are reassuring within the authorized window, but surveillance remains needed for rare outcomes, underrepresented populations, repeat vaccination, coadministration, off-window exposure, and long-term child outcomes. To distinguish precaution from causality, regulatory communication should highlight the difference between a numerical imbalance or safety signal and an established adverse reaction, report absolute event counts and the compatible effect range, and state explicitly when a gestational restriction is precautionary and subject to revision as post-authorization evidence accumulates [9,21,22,23,24,25,26].

## 6. Real-World Evidence for Vaccine Safety Surveillance

### 6.1. Spontaneous Reporting and Individual Case Assessment

Spontaneous reporting systems can rapidly detect unexpected, distinctive, or very rare events, identify temporal patterns, support case-series review, and generate hypotheses after rollout. Pregnancy reports should capture vaccination date, gestational age, product and dose, coadministration, maternal risk factors and infection history, prenatal investigations, pregnancy and neonatal outcomes, and follow-up. Standardized forms and Brighton Collaboration or GAIA definitions improve completeness and comparability [30,53].

Spontaneous reporting is best viewed as a hypothesis-generating tool, not as a basis for incidence estimation or reassurance. Such systems cannot estimate incidence because exposure denominators and reporting probabilities are usually unknown. Underreporting, stimulated reporting, preferential reporting of adverse outcomes, and incomplete follow-up further limit interpretation. Individual causality assessment can clarify diagnosis, timing, and alternatives but rarely establish or exclude a population association. Spontaneous reports should therefore trigger structured signal evaluation rather than independently support reassurance or alarm.

### 6.2. Pregnancy Vaccine Exposure Registries

Pregnancy vaccine exposure registries prospectively enroll exposed pregnancies and follow maternal, fetal, neonatal, and infant outcomes. They are most useful for uncommon or non-routine exposures, confirming product or gestational timing, detailed clinical validation, or variables poorly captured in routine data. They can collect reasons for vaccination, prenatal screening, nonprescription exposures, and detailed congenital-anomaly phenotypes.

Limitations include slow and selective enrollment, loss to follow-up, weak or external comparators, and years of accrual before adequate precision. Before initiation, sponsors and regulators should define the research question, target population, expected exposure, comparator, outcome validation, minimum detectable risk, milestones, stopping criteria, and integration with database evidence. Registries unlikely to answer a clinically relevant question should be replaced or complemented by database or hybrid designs, including linkage to electronic health records or administrative data [30,44].

### 6.3. Linked Maternal-Infant Databases

Linking claims, electronic health records, immunization registries, birth records, congenital anomaly registries, and child development data provides scale for uncommon maternal, fetal, neonatal, and childhood outcomes. The Vaccine Safety Datalink and the US Food and Drug Administration Sentinel System use standardized data structures, validated pregnancy algorithms, maternal-infant linkage, and rapid-cycle analyses for surveillance and hypothesis testing [19,42]. VAC4EU and DARWIN EU enable multinational distributed analyses under local data governance [10,11].

These infrastructures are most informative when prespecified protocols, common definitions, and reusable code are implemented across databases, enabling replication, heterogeneity assessment, and pooled estimates without transferring individual-level data. Size alone does not ensure validity: vaccination could be incomplete, gestational age estimated, maternal-infant linkage unsuccessful, and coding accuracy variable. Studies should validate key algorithms, test alternative definitions, report linkage completeness, and describe populations absent from the data. Long-term offspring surveillance is increasingly being extended through linked population cohorts and dedicated maternal-vaccine cohorts, but follow-up duration, attrition, outcome validation, and postnatal confounding remain central limitations [45,46].

### 6.4. Active and Sequential Vaccine Safety Surveillance

Active and sequential surveillance can evaluate prespecified outcomes during early rollout. Observed-to-expected comparisons, self-controlled analyses, and sequential cohort monitoring may be useful for adverse events of special interest, and rapid-cycle methods are now being applied to maternal RSV vaccination [25,41].

Speed entails trade-offs. Early recipients could differ from later recipients, confounder data could be immature, historical comparators could reflect secular changes, and concurrent unvaccinated groups could differ in healthcare use, infection risk, or antenatal care. Sequential alerts should prompt refinement, record validation, and complementary studies rather than causal conclusions. Protocols should prespecify alert thresholds, repeated-testing adjustments, comparator updates, and transition to formal evaluation.

### 6.5. Distributed Networks and Common-Protocol Implementation

Multidatabase studies do not require central transfer of individual-level pregnancy data. In distributed networks such as VAC4EU and DARWIN EU, data partners can implement harmonized definitions, common data models, and prespecified analytic code locally under each partner’s governance, with aggregate results returned for synthesis. This architecture supports common-protocol replication while respecting privacy, access, and data-use requirements. Nevertheless, permissions, funding, data curation, validation, and local analytic capacity remain study- and network-specific constraints [10,11].

Taken together, these complementary approaches illustrate that confidence in maternal vaccine safety is not established by any single design, but strengthened through the accumulation and triangulation of evidence across the vaccine lifecycle (Figure 1). Important methodological challenges nevertheless remain in interpreting evidence on the safety of pregnancy vaccines. Figure 1 should therefore be read functionally: preclinical evidence addresses biological plausibility, RCTs establish efficacy and common short-term safety, spontaneous reporting detects unexpected signals, pregnancy registries add clinical detail and exposure confirmation, linked databases quantify comparative risks at scale, and active, sequential, and distributed-network analyses support rapid refinement, replication, and generalizability [7,10,11,19,25,30,41,42,44].

## 7. Methodological Challenges in Pregnancy Vaccine Pharmacoepidemiology

### 7.1. Confounding, Indication, and the Healthy-Vaccinee Effect

Vaccination in pregnancy is not randomly assigned. Recipients and nonrecipients may differ in age, comorbidity, parity, obstetric history, healthcare use, socioeconomic position, health literacy, infection risk, and preventive behavior. These differences can produce a healthy-vaccinee effect, although higher-risk individuals may also be preferentially vaccinated. Calendar time and geography further influence vaccine availability, pathogen circulation, recommendations, and obstetric care. Comparator choice should follow the causal question and eligibility criteria. When a clinically credible active comparator exists, and overlap is adequate, it may better align healthcare use and vaccination behavior than an unvaccinated comparator. For a novel maternal vaccine with no credible active comparator, alternatives include a concurrent unvaccinated but vaccine-eligible risk set, calendar-time-matched nonrecipients with matching or weighting, or self-controlled designs. Historical comparators are best reserved for rapid signal contextualization [7,32]. Matching, stratification, or weighting address only measured confounding. Negative-control outcomes or windows, quantitative bias analysis, and replication can help assess residual bias [32].

### 7.2. Gestational Timing and Time-Related Bias

Pregnancy is indexed by gestational age, and vaccine eligibility often begins after a specified week. Pregnancies must remain ongoing and eligible until vaccination. Classifying future recipients as exposed from conception creates immortal-time bias because early losses cannot occur during the pre-vaccination period assigned to the exposed group. Analyses should therefore use time-varying exposure, risk-set sampling, or target trial emulation with aligned eligibility, treatment assignment, time zero, and follow-up [33]. In pregnancy-specific target trial emulation, time zero should be the first point at which both strategies being compared are well-defined and participants satisfy the same eligibility criteria (i.e., often the start of the recommended gestational vaccination window). Last menstrual period can be appropriate when the strategies begin at conception, and first antenatal visit when the target population is explicitly defined at pregnancy recognition. Gestational age may additionally serve as the analysis time scale and an effect modifier, so the estimand should specify both eligibility time and the biologically relevant exposure window [34,35]. Target trial emulation is a design framework for aligning eligibility, exposure assignment, time zero, and follow-up; it does not by itself eliminate residual confounding, exposure or outcome misclassification, or selection bias. These threats require separate confounder measurement, validation, comparator checks, and sensitivity or quantitative bias analyses [7,33,34,35].

The relevant exposure window depends on the outcome. First-trimester exposure is most relevant to structural anomalies; later exposure may be relevant to preterm birth, hypertensive disorders, delivery complications, neonatal adaptation, and antibody transfer. Broad “vaccinated during pregnancy” categories combine biologically distinct windows. Analyses should therefore capture product, dose, gestational age, vaccination-to-delivery interval, coadministration, and maternal infection before or after vaccination.

Gestational timing also determines neonatal antibody exposure through transplacental transfer [54]. Transfer varies with vaccine characteristics, gestational age at vaccination and delivery, interval to birth, prematurity, and maternal conditions such as HIV infection or malaria. Infant analyses should distinguish maternal vaccination from neonatal antibody concentration: vaccination is the intervention, whereas transferred antibodies may mediate protection and modify responses to pediatric vaccines. When available, antibody measures can help explain heterogeneity but require an explicit causal framework.

### 7.3. Conditioning on Pregnancy Recognition and Live Birth

Many databases identify pregnancies only after a clinical encounter or delivery, missing very early losses and potentially linking ascertainment to healthcare contact. Selection can therefore arise before cohort entry: conditioning on survival to pregnancy recognition, a minimum gestational age, or live birth can open noncausal pathways when vaccination, infection, or determinants of the outcome also affect pregnancy survival. Congenital-anomaly studies are particularly vulnerable when terminations after prenatal diagnosis are absent, and the problem is not limited to live-birth-only analyses [13,14,36].

No single method fully resolves these limitations. Studies should describe pregnancy identification, include non-live outcomes where possible, and distinguish exposure before and after pregnancy recognition. When selection into the analytic cohort is measurable, inverse-probability-of-selection weighting can address measured determinants; quantitative bias analysis or bounding can assess structurally unobserved losses or terminations under plausible exposure-survival-outcome relationships. Multiple imputation is appropriate for missing measured covariates or partially observed outcomes under explicit missing-data assumptions, but does not by itself recover pregnancies that never enter the database. Congenital-anomaly studies should compare live-birth-only estimates with analyses incorporating stillbirths and terminations where available and report how large plausible selection bias would need to be to alter the conclusion [13,14,36]. Maternal-infant linkage studies should report linkage rates and compare linked with unlinked records; delayed child outcomes also require accounting for migration, insurance changes, loss to follow-up, and differential healthcare contact.

### 7.4. Outcome Definition, Validation, and Multiplicity

Pregnancy outcomes are heterogeneous. Preterm birth may be spontaneous or medically indicated; hypertensive disorders include distinct clinical entities; and congenital anomalies differ in prevalence, severity, and plausibility. Composite outcomes may increase power but obscure clinically important components. Definitions should therefore be hierarchical, prespecified, and aligned with GAIA or other validated standards [37].

Validation should reflect event frequency, severity, and regulatory importance. Algorithms with high positive predictive value may suffice for common outcomes, whereas rare or serious events may require record review, adjudication, or registry confirmation. Multiple outcomes and risk windows increase the probability of chance findings; studies should prespecify primary outcomes, report planned analyses, distinguish exploratory from confirmatory results, and interpret associations according to plausibility, effect size, precision, negative controls, and replication. For subtype classification, no universal positive predictive value threshold is methodologically valid across settings because acceptable misclassification depends on outcome frequency, expected effect size, and intended use. As a pragmatic benchmark, a validated U.S. vital-record algorithm classified spontaneous and provider-initiated preterm birth with positive predictive values of 85% and 89%, respectively [38]. Where local performance is materially lower or unknown, subtype analyses should be treated as exploratory unless supplemented by chart validation, probabilistic bias analysis, or another explicit misclassification correction.

### 7.5. Triangulation Rather than Methodological Uniformity

Each design has distinct strengths and biases: RCTs reduce baseline confounding but lack statistical power for uncommon outcomes; registries provide clinical detail but often have weak comparators; claims and electronic health records provide scale but limited phenotyping; self-controlled designs address time-invariant confounding only under specific assumptions; and spontaneous reports detect unusual events but cannot quantify risk. Confidence increases when findings converge across designs with different bias structures [5,7].

Triangulation should be planned prospectively. Signal-evaluation plans should specify which rapid analyses will be followed by comparative cohorts, self-controlled studies, record validation, or multinational replication. Discordance should prompt examination of exposure windows, outcome definitions, populations, missing data, and residual confounding. The aim is to explain differing estimates and identify the most credible assumptions, not select the most reassuring result. When estimates disagree, the first step is to determine whether they target the same estimand, population, gestational window, and outcome. Weight should then reflect precision relative to a clinically meaningful threshold, susceptibility to bias, exposure and outcome validation, and independent replication. An underpowered null RCT should not automatically override a well-designed observational excess, while an isolated observational excess should not override a precise RCT without evaluating confounding and misclassification. Regulatory action should be proportional to event seriousness, biological plausibility, and residual uncertainty, with precautionary measures explicitly identified as provisional when causal evidence remains incomplete [7,9].

## 8. A Decision Framework for Pregnancy Vaccine Safety Studies

Study design should be selected for the specific safety question rather than by a fixed evidence hierarchy. Investigators should prespecify the target population, product and dose, outcome frequency, biologically relevant gestational risk window, comparator, clinically meaningful effect size, major anticipated biases, and key sensitivity analyses. Follow-up should match outcome latency, from days for reactogenicity to years for developmental outcomes, and the evidentiary threshold should be linked to the intended regulatory action. Figure 2 depicts iterative evidentiary states—hypothesis generation, evidence generation, refinement, consolidation, confirmation, and benefit-risk decision—while Table 3 translates these principles into practical design elements. Discordant evidence can move the process back to refinement rather than automatically toward reassurance [7,8,9].

## 9. Implications for Regulators, Developers, and Immunization Programs

Regulators should define pregnancy-specific evidence requirements early and align clinical development, pharmacovigilance, and risk management plans. For vaccines intended for pregnancy, core post-authorization studies should specify clinically meaningful risk thresholds, gestational windows, comparators, validation, timelines, and decision rules before approval. Cross-jurisdiction coordination and public registration of protocols and results would improve comparability and transparency.

Developers should implement these requirements using harmonized exposure definitions and standardized outcomes, including GAIA-compatible definitions [37], across RCTs, registries, and database studies, with systematic follow-up of incident pregnancies and maternal-infant linkage where feasible. If a stand-alone registry cannot achieve informative precision promptly, prioritize large database or hybrid studies.

Immunization programs should embed safety surveillance from rollout. Uptake, gestational timing, coadministration, pathogen circulation, and access to antenatal care shape both benefit and observed safety; administration errors, storage, handling, and communication can also affect confidence. Surveillance should link immunization, maternal, pregnancy, birth, neonatal, and pediatric data.

Communication should match evidence maturity and precision. “No signal detected” means no excess was identified within the design and data limits; “no increased risk observed” should include the estimate, confidence interval, and compatible risk range; “risk excluded” should be reserved for evidence with sufficient sample size, follow-up, and replication to rule out a clinically relevant increase. Consistent terminology limits both premature reassurance and avoidable hesitancy.

Global implementation should be tiered to data availability rather than conditioned on mature linked electronic-health-record infrastructure. Where immunization, EHR, and vital-record linkage is unavailable, a minimum maternal-vaccine safety platform can be embedded in antenatal and delivery registers or sentinel hospitals, using standardized exposure fields, GAIA diagnostic-certainty levels, prospective pregnancy follow-up, local background-rate estimation, and targeted chart review. Regional or multinational pooling can then improve precision. Reviews have identified existing perinatal data systems in low- and middle-income countries that can support active surveillance, and GAIA case definitions have been field-tested in South Africa and Gambia [39,40].

This resource-adapted model cannot immediately reproduce the scale or phenotyping of mature linked databases, but it permits locally valid signal detection and progressive strengthening of surveillance capacity. The evidentiary claim should remain proportional to data completeness, outcome validation, and achievable precision.

## 10. Future Directions

Several priorities could strengthen the science of vaccine safety during pregnancy.

First, an internationally agreed core outcome set should distinguish acute maternal events, obstetric outcomes, fetal outcomes, neonatal outcomes, and delayed childhood outcomes while preserving clinically meaningful subtypes. For example, spontaneous and medically indicated preterm birth should not be combined when their causes and clinical implications differ.

Second, healthcare systems should share and evaluate validated algorithms for pregnancy onset, gestational age, non-live outcomes, vaccine exposure, and maternal-infant linkage because performance may not translate across settings. Artificial intelligence may support case triage, phenotyping, deduplication, and signal prioritization across heterogeneous data, but pregnancy-specific use raises risks of algorithmic bias, differential outcome misclassification, poor transportability, false-positive signal generation, limited explainability, and data-governance or privacy constraints. AI-generated alerts should therefore remain prioritization signals rather than causal findings: document model versioning, calibration, threshold prespecification, external validation, human oversight, and conventional epidemiologic validation before drawing regulatory conclusions [55,56].

Third, regulators and research networks should develop modular master protocols that can be rapidly activated for new maternal vaccines or emerging pathogens. These protocols should combine rapid-cycle surveillance, comparative cohort studies to refine signals, standardized outcome validation, and long-term maternal-child follow-up. In federated networks, they should specify a common minimum data model, version-controlled analytic code, and reporting standards, while sustainable data curation, validation, coordination, and local analytic capacity remain implementation requirements [10,11].

## 11. Conclusions

Pregnancy vaccine safety requires coordinated evidence across the immunization lifecycle, not reliance on a single design. RCTs remain essential for efficacy, immunogenicity, reactogenicity, and common short-term outcomes, while pharmacovigilance, registries, linked comparative data, and longitudinal maternal-child follow-up address rarer and delayed outcomes. Across designs, product- and gestational-age-specific exposure definitions, credible comparators, validated outcomes, appropriate risk windows, and transparent reporting of residual uncertainty are essential. The intensity of evidence generation should remain proportional to anticipated pregnancy exposure, clinical need, and available infrastructure [6,7,8,39,40].

## Figures and Tables

**Figure 1:**
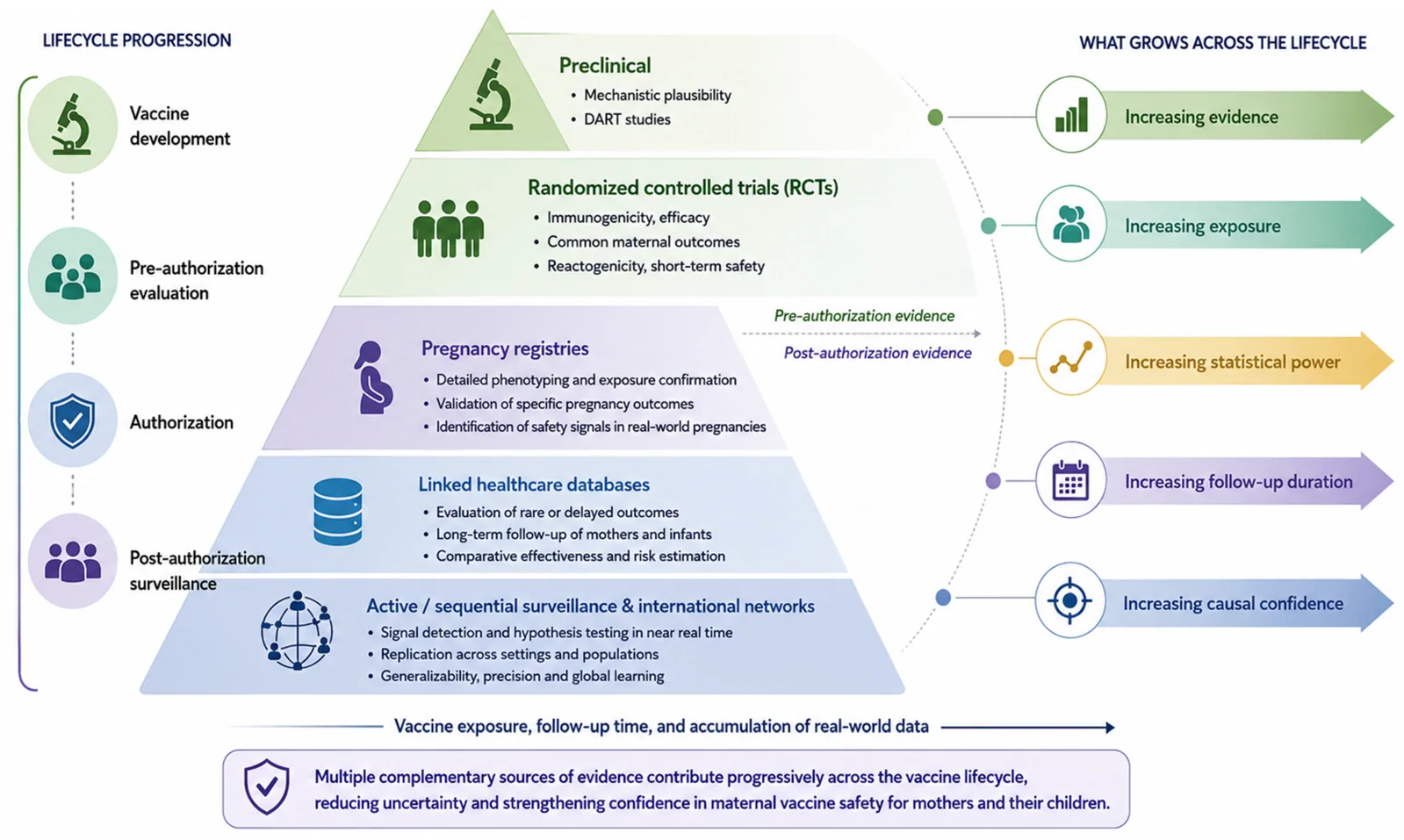
Authors’ proposed integrated lifecycle evidence architecture for maternal vaccine safety assessment, informed by ICH/EMA guidance and complementary methodological literature [7,8,9]. The relative contribution of each design changes with exposure, follow-up, and the safety question; the figure is a methodological synthesis rather than a regulatory hierarchy.

**Figure 2:**
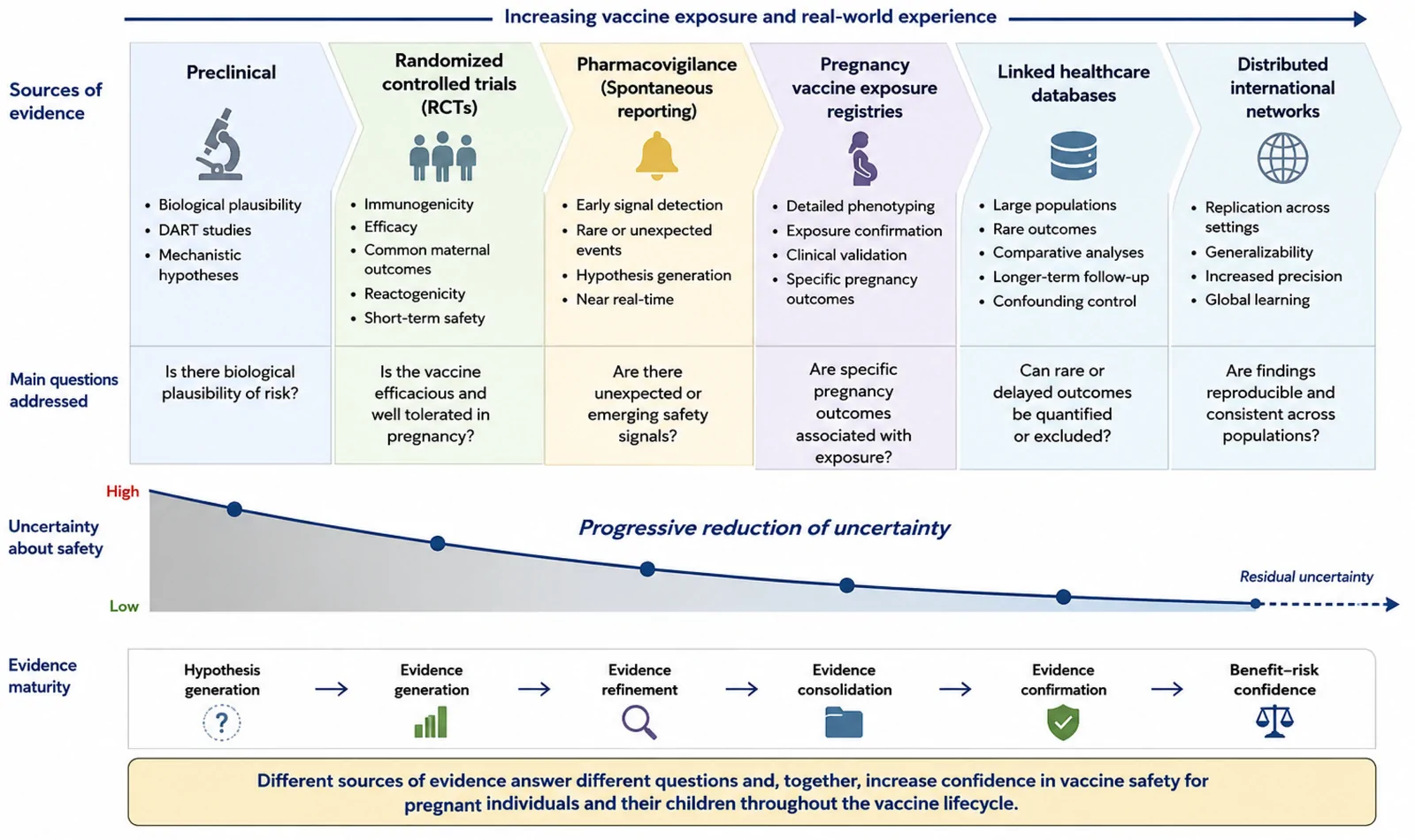
Authors’ proposed decision pathway for progressive reduction in uncertainty through complementary evidence generation across the vaccine lifecycle, informed by regulatory and methodological principles [7,8,9].

**Table 1:** What pregnancy RCTs can establish, what they cannot establish alone, and the evidence needed to estimate residual uncertainty.

Safety Question	What RCTs Contribute	What RCTs Cannot Establish Alone	Complementary Evidence
Efficacy, immunogenicity, reactogenicity, common short-term outcomes	Randomization supports causal estimates with standardized follow-up [6,21,27,28]	Rare imbalances remain imprecise, external validity may be limited	Active surveillance and linked comparative cohorts
Obstetric and neonatal outcomes	Can estimate common outcomes and identify large imbalances	Usually underpowered to exclude modest increases in uncommon outcomes	Linked maternal-infant cohorts and sequential surveillance [17,18,19,23,24,25,26]
Pregnancy loss and congenital anomalies	Can generate signals when events occur during trial follow-up	Specific phenotypes and non-live outcomes are often too sparse for precise inference	Population-based pregnancy/congenital-anomaly data and exposure registries [12,13,14,20,30,31]
Delayed infant or childhood outcomes	Can standardize early infant endpoints	Typical trial duration and attrition limit delayed-outcome assessment	Long-term maternal-child linkage and multinational common-protocol studies [10,11,19]

**Table 2:** Authors’ synthesis of principal methodological challenges in pregnancy vaccine pharmacoepidemiology and practical responses, informed by current methodological and regulatory literature.

Challenge	Why Inference Can Be Biased	Recommended Current Response	Implementation Priority
Confounding and healthy-vaccinee effects	Uptake differs by healthcare use, baseline risk, access, and preventive behavior	Use an active comparator only when clinically credible; otherwise use an eligible concurrent nonrecipient risk set, matching/weighting, negative controls, and quantitative bias analysis [7,32].	Improve capture and harmonization of behavioral, socioeconomic, infection, and healthcare-use determinants
Gestational timing and immortal-time bias	Eligibility and exposure status change during pregnancy	Use time-varying exposure or risk-set approaches and align eligibility, treatment assignment, time zero, and follow-up; consider gestational age as the analysis time scale/effect modifier. Target trial emulation addresses this alignment but does not eliminate residual confounding, misclassification, or selection bias [7,33,34,35].	Implement target trial specifications for gestational eligibility rules
Pregnancy recognition and live-birth selection	Early losses, terminations, or stillbirths may be absent or differentially observed	Include non-live outcomes where possible; use inverse-probability-of-selection weighting for measured selection and quantitative bias analysis or bounding for structurally unobserved outcomes; compare alternative pregnancy-identification strategies where feasible [13,14,36].	Strengthen linkage to prenatal diagnosis, fetal-death, and congenital-anomaly sources
Outcome misclassification and multiplicity	Administrative codes may not capture clinically meaningful subtypes; multiple windows increase chance findings	Use validated algorithms, chart review/adjudication for key outcomes, prespecification, and sensitivity analyses for misclassification and multiplicity [37,38].	Develop reusable validation libraries and cross-setting evaluation of GAIA-compatible definitions
No credible active comparator	Novel maternal vaccines may have no alternative vaccine with the same indication and timing	Do not force an active comparator; use a concurrent vaccine-eligible unvaccinated group, self-controlled designs when assumptions are met, and observed-to-expected analyses primarily for signal contextualization [7,15,32].	Prespecify a comparator hierarchy and switching rules before rollout
Network heterogeneity and resource constraints	Data capture, linkage, coding, governance, and analytic capacity vary across settings	Use common protocols/data models under local governance; where linkage is limited, use sentinel sites, prospective registers, local background rates, and targeted validation [10,11,39,40].	Invest in local validation, sustainable funding, and federated or regional analyses to improve precision

**Table 3:** Authors’ proposed mapping of pregnancy vaccine safety questions to evidence strategies and practical design elements.

Safety Question	Most Informative Evidence Strategy	Practical Design Elements	Interpretation
Common maternal reactions, immunogenicity, efficacy, and gestational-window optimization	Pregnancy-specific RCTs [6,21,27,28]	Frequency: common. Comparator: randomized placebo/control or an active control when scientifically justified. Window: prespecified acute reactogenicity and gestational strata. Main bias: attrition/nonadherence and limited external validity. Sensitivity analyses: intention-to-treat and per-protocol where appropriate; gestational-stratum analyses; missing-outcome assumptions.	Provides causal estimates for common and short-term outcomes; uncommon imbalances require further evaluation
Emergence of an unexpected or (very) rare event	Spontaneous reporting, case review, observed-to-expected analyses, and rapid-cycle surveillance [7,15,16,25,41]	Frequency: very rare or unknown. Comparator: gestational-, calendar-, and population-specific background rates for observed-to-expected analyses; spontaneous reports have no causal comparator. Window: prespecified biologically plausible risk interval. Main bias: under- or stimulated reporting, denominator uncertainty, and multiplicity. Sensitivity analyses: alternative background rates and windows; record validation; repeated-testing adjustment.	Supports signal detection and prioritization, but not causal or incidence estimation from spontaneous reports alone
Comparative risk of obstetric or neonatal outcomes	Linked healthcare and registry cohorts, including target trial emulation where feasible [7,17,18,19,20,32,33,34,35,42,43]	Frequency: uncommon to common. Comparator: concurrent vaccine-eligible nonrecipient; active comparator only when clinically credible. Window: outcome-specific, aligned from eligibility/time zero through delivery or neonatal follow-up. Main bias: residual confounding, healthy-vaccinee effects, time-related bias, and misclassification. Sensitivity analyses: alternative comparator/propensity-score specifications, negative controls, quantitative bias analysis, and alternative exposure/outcome definitions.	Estimates relative and absolute risks but remains vulnerable to residual confounding and misclassification
Pregnancy loss, congenital anomalies, or uncommon and inadvertent exposures	Population-based pregnancy and congenital-anomaly data, complemented by exposure registries [12,13,14,30,31,36,44]	Frequency: uncommon overall and often rare by phenotype. Comparator: concurrent internal comparison including non-live outcomes where possible. Window: periconception/first trimester for structural anomalies; conception-to-loss for pregnancy loss. Main bias: pregnancy-recognition/live-birth selection, exposure-timing error, and anomaly ascertainment. Sensitivity analyses: include stillbirths/terminations when available; selection weighting or bounding; phenotype-specific and alternative pregnancy-identification analyses.	Requires large populations; registries add clinical detail but rarely provide sufficient comparative reassurance alone
Delayed childhood outcomes and confirmation of signals	Long-term maternal-child linkage and multinational common-protocol studies/PASS [7,10,11,19,45,46]	Frequency: generally uncommon and heterogeneous. Comparator: concurrent mother-child comparisons, independent replicated cohorts, and sibling/familial controls when appropriate. Window: prespecified long-term follow-up matched to outcome latency. Main bias: attrition/linkage failure, time-varying postnatal confounding, and outcome misclassification. Sensitivity analyses: linked-versus-unlinked comparisons, inverse-probability-of-censoring, negative controls, alternative outcome definitions, and independent replication.	Evaluates delayed outcomes and strengthens inference through replication across populations and designs

Abbreviations: PASS: Post-authorization safety studies; RCT: randomized controlled trial.

## Data Availability

No new data were created or analyzed in this study. Data sharing is not applicable to this article.
